# Mpox in sports: A comprehensive framework for anticipatory planning and risk mitigation in football based on lessons from COVID-19

**DOI:** 10.5114/biolsport.2024.144014

**Published:** 2024-10-10

**Authors:** Karim Chamari, Helmi Ben Saad, Wissem Dhahbi, Jad Adrian Washif, Abdelfatteh El Omri, Piotr Zmijewski, Ismail Dergaa

**Affiliations:** 1 Research & Education, Naufar, Wellness and Recovery Center, Doha, Qatar; 2 Heart Failure Research Laboratory (LR12SP09), Farhat Hached Hospital, Faculty of Medicine of Sousse, University of Sousse, Tunisia; 3 High Institute of Sport and Physical Education of El Kef, University of Jendouba, El Kef, Tunisia; 4 Qatar Police Academy, Police College, Training Department, Doha, Qatar; 5 Sports Performance Division, Institut Sukan Negara Malaysia (National Sports Institute of Malaysia), Kuala Lumpur, Malaysia; 6 Surgical Research Section, Department of Surgery, Hamad Medical Corporation, Doha 3050, Qatar; 7 Institute of Sport - National Research Institute, Warsaw, Poland; 8 Higher Institute of Sports and Physical Education of Ksar Said, University of Manouba, Manouba, Tunisia

**Keywords:** Athlete health, Contact, Epidemiology, Event management, Infection control, Infectious disease, Monkeypox, Mpox, MPXV, Orthopoxvirus, Pandemic preparedness, Risk assessment, Sports medicine

## Abstract

The World Health Organization’s declaration of mpox (formerly known as monkeypox) as a public health emergency of international concern (PHEIC) in July 2022, followed by its resurgence in 2024, has sparked concerns about its potential impact on sports, especially contact sports such as football. Although mpox is not a pandemic (as of late September 2024), the coronavirus disease 2019 (COVID-19) experience offers valuable lessons for proactive planning in sports. Our conceptual framework has been designed to draw insightful lessons from the COVID-19 pandemic to assist sports organizations in planning for and preventing similar situations. We aimed to draw lessons from COVID-19 to help sports organizations enhance practical preparedness through effective planning and mitigation strategies. Accordingly, we sought to assess the potential impact of mpox on sports, with a focus on football (soccer), and to develop strategies for prevention, management, and preparedness based on epidemiological insights and lessons from COVID-19 pandemic experience. Here we review mpox’s pathophysiology and possibility of transmission in sports settings and discuss tailored strategies, including risk assessments, testing protocols, hygiene measures, and return-to-play policies. This review highlights key differences between mpox and COVID-19 in transmission, incubation, and management, emphasizing the need for customized prevention and control measures in sports. We propose innovative risk assessment methods using global positioning system tracking and machine learning for contact analysis, alongside tailored testing and hygiene protocols. We emphasize the importance of proactive planning, noting improved preparedness in the sports community compared to the early days of COVID-19. In conclusion, our proposed framework provides sports organizations with practical tools to manage potential risks associated with mpox, ensuring the continuity of activities while prioritizing public health.

## INTRODUCTION

Mpox (previously known as monkeypox) is a disease that can be transmitted between animals and humans [1, 2]. It is caused by a specific virus called monkeypox virus (MPXV), which belongs to the Orthopoxvirus genus in the Poxviridae family [1]. In humans, the disease typically has an incubation period of 5–21 days and subsequently advances through two distinct phases (the prodromal phase and the rash phase, with the latter characterized by evolving skin lesions) [1, 3]. In May 2022, the World Health Organization (WHO) reported an occurrence of human mpox in multiple nations that had previously been free of the disease [4]. According to Centers for Disease Control and Prevention data (dated March 1, 2023), there have been 86,231 confirmed cases of mpox and 105 deaths reported from 110 countries and territories worldwide [5]. Importantly, over 90% of these nations were reporting mpox for the first time [6]. As of September 22, 2024, the total number of lab-confirmed cases of mpox has risen to 106,310 (234 total lab-confirmed deaths and 123 countries reporting cases) [7]. A phylogenetic study indicated that this outbreak was linked to the virus originating from the West African clade [8]. Phylogenetic studies have identified two distinct clades of MPXV: the Congo Basin clade and the West African clade, with the latter being responsible for the 2022 global outbreak [8]. Nevertheless, most of the individuals in this outbreak did not have any proof of having travelled to locations in Central or West Africa, where MPXV is commonly found [9]. The main factor contributing to this outbreak in humans was the spread of the virus through close physical contact among males who engage in sexual activity with other males [9]. The shifting epidemiology of mpox has prompted concerns regarding its increasing transmission in non-endemic nations and the pressing necessity to manage and avert its spread.

On August 14, 2024, the WHO declared that “the rise in mpox cases constitutes a public health emergency of international concern (PHEIC)” [6]. The transmission of mpox between humans primarily occurs through close physical contact, respiratory droplets, and contaminated objects [10–13]. The virus can spread through direct contact with skin lesions, bodily fluids, or respiratory secretions of infected individuals [11–14]. Indirect transmission via contaminated materials such as bedding, clothing, or shared objects is also possible [11–13, 15]. The prolonged incubation period of up to 21 days poses significant challenges for containment efforts [16]. During this relatively long period, infected individuals may unknowingly spread the virus, complicating contact tracing and early intervention strategies [16]. This extended asymptomatic period increases the risk of widespread transmission before symptoms become apparent [16]. The latter is particularly concerning in some specific environments, e.g. the sporting environment, where athletes are typically in contact with each other on a daily basis, let alone the crowds that gather to attend sporting competitions. Indeed, research has highlighted the potential for asymptomatic or pre-symptomatic transmission of mpox, which could have significant implications for sports settings [17]. The rising transmission rates of mpox are of growing concern for the sports world. Football, as a high-contact sport with global appeal, serves as an ideal case study for examining the potential impact of mpox on sports. The frequent physical interactions between players, shared equipment, and mass gatherings associated with football events can create unique challenges for disease prevention and control [17]. While, as of late September 2024, it’s less infectious than coronavirus disease 2019 (COVID-19), the potential for viral mutations [18] and increased infectivity could have severe implications for athletic activities [19]. The transmission methods of mpox are particularly worrisome in sports contexts. Skin-to-skin contact, common in contact sports and among some spectators, presents a higher risk compared to the primarily respiratory transmission of COVID-19, and mass gathering events, both for athletes and fans, and creates environments conducive to viral spread [17]. Unlike COVID-19, where wearing face masks provided some protection, the skin contact transmission route of mpox makes such measures ineffective [17].

The sports community faces unique challenges in addressing the mpox threat. While lessons learned from COVID-19 could provide valuable insights, their applicability may be limited due to the differing transmission mechanisms of mpox [17]. Contact sports, in particular, might face significant risks due to the frequent skin-to-skin contact between athletes. Although vaccines show promise, vaccine hesitancy remains high, partly due to concerns stemming from COVID-19 vaccine side effects [20]. The potential for a mpox pandemic raises questions about the feasibility and impact of lockdowns on sporting events and athlete training regimens [21].

To the best of the authors’ knowledge, this study presents the first comprehensive conceptual framework for understanding the potential impact of mpox on the sports world. Addressing the identified research gaps, including the lack of sport-specific transmission data, limited understanding of the virus’s behaviour in the athletic environments, and absence of tailored prevention strategies for sports settings, our conceptual study aims to: *(i)* analyse the pathophysiology and transmission mechanisms of mpox in sports environments, with a particular emphasis on football as a representative of high-contact sport; *(ii)* develop and evaluate tailored epidemiological strategies for sports, including risk assessment, testing protocols, hygiene measures, return-to-play policies, and spectator management; *(iii)* examine the potential impact of mpox on sports activities, encompassing physical health, performance considerations, and implications for events and training regimens; and *(iv)* assess the public health implications and propose preparedness strategies for managing potential pandemics in sports settings, drawing on experiences from the COVID-19 pandemic.

## PATHOPHYSIOLOGY AND TRANSMISSION OF MPOX

Mpox follows a complex pathophysiological progression [13]. The disease course can be divided into three distinct phases: *i)* incubation (lasting 5–21 days), *ii)* prodromal (1–5 days), and *iii)* exanthem (2–4 weeks) [22]. The prodromal phase is characterized by systemic symptoms including fever, malaise, myalgia, and lymphadenopathy [22]. The exanthem phase involves the development of a characteristic rash, progressing from macular lesions to papules, vesicles, and pustules before crusting and desquamation [23]. This prolonged disease course and the potential for asymptomatic transmission create potential challenges challenges for containment in sports settings.

The virus spreads primarily through close contact with infected persons, animals, or contaminated materials, entering the body via broken skin, the respiratory tract, and/or mucous membranes [24]. Football, as the world’s most popular sport, with extensive global participation and spectatorship, serves as an ideal model for examining the potential impact of mpox in sports settings. Its widespread practice, high-contact nature, and the lessons learned from COVID-19 management in football provide a comprehensive framework for analysis [25]. Importantly, transmission can occur through skin-to-skin contact even before visible lesions appear, significantly increasing the risk in sports settings. In football, the frequent close physical contact during tackles, aerial duels, or on-field celebrations could potentially facilitate virus transmission, even in asymptomatic cases [26]. This risk extends to other contact sports, including combat (e.g. boxing, karate, silat, and wrestling) and team (e.g. rugby, hockey, volleyball, and basketball) sports with similar levels of physical interaction [26].

**FIG. 1:**
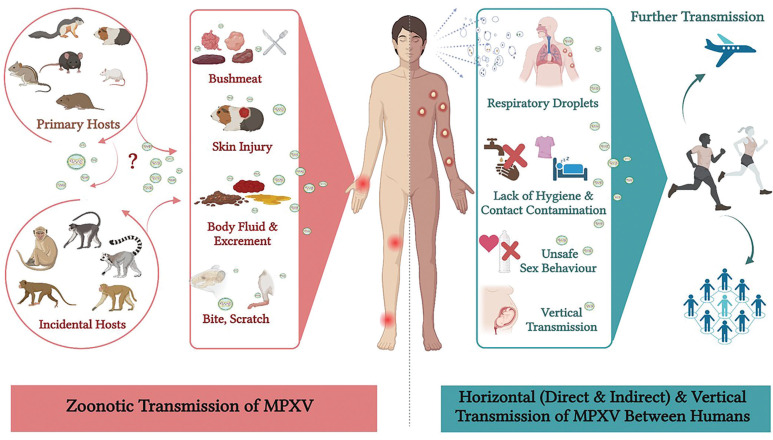
Zoonotic and human-to-human transmission of mpox virus (MPXV) (created with Biorender.com) [11-13]. This figure illustrates the transmission pathways of mpox, including both zoonotic and human-to-human routes. Zoonotic transmission occurs when humans encounter infected animals (primarily rodents and primates) through direct interaction, animal bites, scratches or exposure to bodily fluids, as well as through the consumption of contaminated bushmeat. Human-to-human transmission can be categorized into horizontal and vertical routes. Horizontal transmission includes skin-to-skin contact, exposure to contaminated materials, and inhalation of respiratory droplets. Vertical transmission refers to the transfer of the virus from mother to fetus. The understanding of these transmission pathways is crucial for the development and implementation of effective prevention and control strategies (see Figure 2).

Direct physical contact with skin, regardless of visible lesions, or bodily fluids of an infected individual is a key transmission route. This applies to football, where players frequently come into close physical contact during matches and training sessions. The risk then extends beyond obvious skin lesions to include contact with seemingly healthy skin of infected individuals [27]. Moreover, MPXV can persist on surfaces and objects, creating the potential for indirect transmission [28]. In football, shared equipment such as balls, training bibs, or even goalposts could become vectors for the virus, emphasizing the need for rigorous cleaning protocols in training facilities and stadiums. Figure 1 summarizes the various transmission pathways of MPXV including zoonotic transmission from animals to humans, and both horizontal and vertical transmission between humans.

While both mpox and COVID-19 are contagious diseases, their transmission mechanisms differ substantially [29]. COVID-19 spreads primarily through respiratory droplets and aerosols, with very few prospective risks in the sports environment [30], whereas mpox relies more on direct physical contact, including skin-to-skin contact without visible symptoms [31]. This distinction is crucial for developing targeted prevention strategies in football and other sports environments. Notably, the COVID-19 pandemic caught the sports world largely unprepared, as there was no recent precedent for such a global health crisis in early 2020 [32]. In contrast, we expect that the experience gained from managing COVID-19 has better equipped sports organizations and policymakers to respond to the potential threat of mpox [32, 33]. Table 1 provides a detailed comparison of COVID-19 and mpox, highlighting key differences relevant to transmission, management, and preparedness in sports settings.

**TABLE 1:** Comparison of coronavirus disease 2019 (COVID-19) and mpox in sports settings.

Characteristic	COVID-19	Mpox
Primary transmission	Respiratory droplets and aerosols	Direct physical contact, including skin-to-skin
Incubation period	2-14 days (average 5-6 days)	5-21 days (average 7-14 days)
Infectiousness	1-2 days before symptom onset	Primarily after symptom onset, but possibly before rash appears
Main symptoms	Fever, cough, fatigue, loss of taste/smell	Fever, rash, lymphadenopathy
Risk in sports	High in indoor, close-contact settings	High in all contact sports, including outdoor
Surface transmission	Possible, but not primary route	More significant risk from contaminated items
Asymptomatic spread	Common	Less common, but possible before the rash appears
Vaccination	Widely available No sport-specific protocols	Limited availability No sport-specific protocols yet (Late September 2024)
Isolation period	Typically, 5-10 days from symptom onset	Until all lesions have crusted and fallen off (2-4 weeks)
Impact on sports events	Widespread cancellations and adaptations	Limited impact so far (Late September 2024), but potential for significant disruption
Prior pandemic experience	No recent precedent, the sports world unprepared	COVID-19 experience should better inform preparedness and decision-making
Risk of mutation	High, led to multiple variants of concern	Low, but the potential exists for increased transmissibility
Policy readiness	Policies developed reactively	Opportunity for proactive policy development and preventative measures mechanisms implementation based on COVID-19 experience

The incubation period for mpox ranges from 5 to 21 days, with most cases showing symptoms within 7 to 14 days [34]. This extended period creates challenges for containment, as players or staff may unknowingly spread the virus before any symptoms or skin lesions appear [34]. These factors complicate the management of sporting events, tournaments, and training schedules across all contact sports, necessitating careful monitoring and preventive measures even for seemingly healthy individuals [35]. While our analysis focuses on football as a representative example, the principles and challenges discussed apply to a wide range of sports, particularly those involving close physical contact [36]. For football clubs and organizations, this approach emphasizes the importance of comprehensive prevention strategies that account for asymptomatic transmission, alongside adaptive measures to mitigate the potential spread of mpox while maintaining competitive activities.

## EPIDEMIOLOGICAL CONSIDERATIONS IN SPORTS

### Risk assessment and contact analysis

In football, the close-contact nature of play presents unique challenges for controlling MPXV transmission. To address this, we can adapt advanced epidemiological tools and integrate them with existing sports technology [37]. Enhanced global positioning system tracking systems, augmented with proximity sensors and biometric data, can create detailed, multi-dimensional contact matrices that account for the intensity, duration, and physiological context of player interactions [38]. These matrices can be analysed using machine learning algorithms and social network analysis to identify potential transmission hotspots and “super-spreader” positions. Machine learning algorithms can play a dual role: first, in research settings to analyse and identify patterns of transmission in sports environments, and second, in the development of systems that can be integrated into competitive games and tournaments [37]. These systems could be used for real-time monitoring of player interactions, helping to identify potential transmission hotspots during matches and practices, thereby enabling more responsive and targeted interventions [37]. The SEIRS-V (for susceptible-exposed-infected-recovered-susceptible-vaccinated) model is an epidemiological compartmental model that includes vaccination status, allowing for more nuanced predictions in partially immunized populations [39]. A football-specific SEIRS-V model, incorporating factors such as varying transmission rates between training and matches, player positions, and crowd presence, can be developed to estimate position-specific and situation-specific R0 values [40]. Environmental sampling for fomite transmission risk can be integrated into spatiotemporal models.

### Testing protocols and symptom monitoring

Mpox testing and monitoring in football should follow a tiered, risk-based approach triggered by specific epidemiological indicators. Initial efforts should focus on enhanced symptom vigilance and basic skin examinations by trained medical staff. If mpox cases are reported in the general population or sports communities, a more intensive protocol should be activated [41]. Mpox infection diagnosis relies on both direct and indirect methods. Nucleic acid amplification tests, particularly reverse transcription polymerase chain reaction (RT-PCR), are widely used for direct detection of viral DNA sequences [41]. In resource-limited settings, pooled sample RT-PCR offers a cost-effective surveillance approach, building on the widespread availability of RT-PCR technology from the COVID-19 pandemic. Indirect tests, which detect the patient’s immune response, include lateral flow assays (LFAs) for detection of Orthopoxvirus immunoglobulins M and G. However, these tests have limitations, such as undetected convalescent samples and cross-reactivity with poxvirus vaccines [41, 42]. While LFAs are rapid and suitable for point-of-care use, they are less accurate than laboratory-based methods such as enzyme-linked immunosorbent assay (ELISA) and chemiluminescent immunoassay (CLIA). ELISA and CLIA offer higher sensitivity and specificity for antibody and antigen detection but require specialized laboratory infrastructure and low-temperature logistics, which may be challenging in low-resource countries [41]. LFAs for mpox antigen detection show promise as a low-cost, rapid alternative but require rigorous validation before widespread implementation in public health surveillance and diagnosis [41, 42].

When handling mpox specimens in the laboratory, strict biosafety measures are essential to prevent accidental exposure and contamination [43]. Samples from identical lesion types can be combined, but those from different types should be kept separate. It is beneficial to gather various lesion types from multiple sites simultaneously. In addition to skin lesions, swabs from the oropharynx, anus, and rectum can be used to detect mpox. However, oropharyngeal results should be interpreted with caution due to limited clinical data [44].

This could include periodic random sampling using polymerase chain reaction (PCR) tests on skin lesion samples or throat swabs from a subset of players and staff [45], prioritizing those with higher risk exposures. Serological surveys might be conducted quarterly to assess team immunity levels, detecting antibodies against mpox [46]. Advanced techniques such as artificial intelligence (AI)-powered dermatological imaging or CRISPR (for clustered regularly interspaced short palindromic repeats)-based diagnostics would only be deployed if cases were confirmed within the football community or if there was a significant outbreak in the surrounding area [47]. This adaptive strategy will ensure that resources are used efficiently, scaling up monitoring efforts only when epidemiological data justify increased vigilance, thus balancing cost-effectiveness with comprehensive disease surveillance.

### Hygiene measures and surface decontamination

To address the prolonged surface persistence of MPXV in football environments, a multi-layered decontamination strategy is essential. High-touch areas and shared equipment should be prioritized, using EPA (environmental protection agency)-registered disinfectants effective against enveloped viruses [48]. Ultraviolet C irradiation systems could be installed in locker rooms and training facilities for overnight sanitization [49]. For textiles, implementing rigorous laundry protocols with high-temperature washing and the addition of virucidal laundry sanitizers is crucial. To reduce reliance on frequent cleaning, antimicrobial surfaces such as copper alloys could be strategically implemented in high-contact areas. For medical staff and players, a risk-stratified approach to personal protective equipment (PPE) should be adopted, with standard precautions for routine interactions and enhanced protection, including respirators, for close-contact treatments or when handling potentially infectious materials [50].

A decontamination strategy for sports facilities in low-resource countries should include regular disinfection of high-touch surfaces, education on hygiene practices, and tracking cards to monitor cleaning schedules. Providing basic protective equipment and raising awareness about proper disposal of contaminated materials are also crucial for effective implementation. Table 2 outlines decontamination strategies for sports facilities that are applicable in all settings, including resource-constrained countries [51–60].

**TABLE 2:** Inclusive decontamination strategies for indoor sports facilities [51-60].

Strategy	Details
Enhance ventilation	Open windows/doors Use low-cost fans Improve air circulation Increase heating, ventilation, and air conditioning filter changes
Establish equipment sanitization protocols	Create schedules for cleaning shared equipment using affordable disinfectants with clear instructions
Install handwashing stations and/or hand sanitizers	Place stations at key locations Use portable stations with foot operation if water is limited
Use disinfectant mats at entrances	Install durable mats for footwear decontamination Regularly replace the disinfectant solution
Manage facility capacity	Implement staggered scheduling Occupancy limits Extend hours to reduce crowding
Implement proper waste management	Place labelled, covered bins for waste Regularly empty and sanitize receptacles
Create designated “clean” and “used” areas	Implement a system for collecting, cleaning, and redistributing shared items like towels Encourage personal hygiene behavior
Choose easily cleanable surfaces	Opt for smooth, non-porous equipment surfaces or apply cleanable coatings
Conduct regular staff training and periodic assessments	Provide training on cleaning, personal protective equipment usage, and safety protocols, with periodic updated courses Regularly audit decontamination measures and seek feedback for improvements
Display clear, multilingual signage	Use graphic-based instructions for hygiene protocols Place signs in key areas
Utilize ultraviolet-C light disinfection	Use ultraviolet-C light for small items or hard-to-clean areas if budget allows
Develop a contact tracing system	Maintain facility usage logs and implement a check-in process for outbreak management

### Gradual return to play and adaptive policies

A phased return-to-play protocol, similar to those used after COVID-19 [26, 61], but tailored to mpox’s longer incubation period (up to 21 days), might be necessary. This approach could involve extended quarantine periods for exposed individuals and a gradual reintegration process monitored by sports medicine professionals. Policies should be adaptive, potentially utilizing Bayesian decision-making frameworks that can quickly incorporate new epidemiological data. Mpox’s longer incubation period necessitates a carefully structured return-to-play protocol in football [62]. This protocol should be risk-stratified, considering the player’s exposure level, vaccination status, and team outbreak situation. A minimum 21-day observation period for exposed individuals is advisable, with a gradual reintegration process involving staged increases in training intensity and group size [63]. A multidisciplinary team including infectious disease specialists and sports medicine professionals should oversee this process. To ensure flexibility, the protocol could employ a Bayesian decision-making framework, allowing real-time adjustments based on new epidemiological data, testing results, and player health metrics [64].

### Spectator management strategies

Unlike COVID-19, where respiratory transmission was the primary concern, mpox poses additional challenges due to potential skin-to-skin transmission. Social distancing measures in stadiums might need to be more stringent, potentially utilizing hexagonal or honeycomb seating arrangements to maximize interpersonal distance [12]. Entry screening could include visual skin checks or thermal imaging to detect fever. Stadiums might need to implement enhanced ventilation systems with HEPA filtration and ultraviolet sterilization to reduce airborne transmission risk. Spectator management for mpox in football requires innovative approaches beyond those used for COVID-19 [65]. Given the risk of skin-to-skin transmission, stadium seating could be redesigned using algorithmic models to maximize interpersonal distance while maintaining capacity. Digital ticketing systems could be used to create “social bubbles” allowing groups that regularly interact to sit together while maintaining distance from others. Recent studies have shown the effectiveness of social bubbles in reducing disease transmission [66]. Entry protocols might include non-invasive screening methods such as AI-powered visual skin anomaly detection, complemented by thermal imaging. To mitigate fomite transmission, stadiums could implement contactless technologies for all fan interactions, from entry to concessions. Environmental controls should focus on both air and surface management, potentially incorporating photocatalytic oxidation systems for continuous disinfection [67]. Fan education campaigns, emphasizing the unique transmission routes of mpox, would be crucial for compliance with these measures. While we propose various technologically advanced solutions, it is important to acknowledge that implementation may be challenging or impossible for many countries due to financial constraints or infrastructure limitations [67]. These suggestions represent ideal scenarios and should be adapted based on local resources and capabilities.

### Data collection and analysis

Implementing a comprehensive syndromic surveillance system across leagues could provide early warning of potential outbreaks [68]. This approach could involve real-time data integration from multiple sources including team medical staff, local health authorities, and even crowd-sourced symptom tracking apps. Advanced machine learning algorithms could be employed to detect anomalous patterns indicative of emerging clusters [68]. A football-specific syndromic surveillance system for mpox would integrate diverse data streams to detect outbreaks early. This system could combine traditional epidemiological data with sport-specific metrics. While not directly related to mpox transmission, changes in player performance, training load tolerance, and recovery times could potentially serve as early indicators of subclinical infection or exposure [69]. Machine learning algorithms, particularly anomaly detection models, could be trained to identify subtle patterns indicative of potential mpox spread. The system could incorporate genomic surveillance, tracking viral mutations that might affect transmissibility or virulence within the football community [70]. To enhance sensitivity, a network of sentinel sites could be established across different leagues and levels of play. This approach would not only serve as an early warning system but also contribute to our understanding of mpox transmission dynamics in high-performance athletic settings [71]. The surveillance system should be designed with data privacy in mind, using secure, anonymized data sharing protocols to protect player and team confidentiality while still allowing for an effective public health response.

### International coordination

Given the potential for cross-border transmission, establishing a global football-specific health information system could be vital. This could function similarly to the WHO’s global outbreak alert and response network, but tailored to the specific needs and rapid movement patterns of international football [72]. A global football-specific health information system for mpox would need to address the challenges presented by the sport’s international nature. This system could leverage existing football networks such as the Fédération Internationale de Football Association (FIFA) and continental confederations to create a rapid alert mechanism for potential outbreaks [73]. It should incorporate real-time travel data of teams and officials, allowing for quick contact tracing across borders. The system could utilize blockchain technology to ensure secure, transparent sharing of health data while maintaining player privacy. A standardized risk assessment tool, tailored to football-specific scenarios, could be integrated to help decision-makers evaluate the safety of international matches and tournaments [74]. This global system should also facilitate rapid sharing of best practices and emerging research relevant to mpox in sports settings [75]. To ensure effectiveness, it should be designed with input from epidemiologists, sports medicine experts, and football governing bodies, creating a collaborative approach to global health security in football.

### Mental health support

The psychological impact of another disease outbreak could be significant. Implementation of regular mental health screenings using validated tools such as the Athlete Psychological Strain Questionnaire could be beneficial [76, 77]. Telepsychiatry services could be made readily available to players and staff. Addressing the mental health implications of an mpox outbreak in football requires a proactive, multifaceted approach. Beyond regular screenings, a comprehensive mental health strategy could include the development of a sport-specific psychological resilience training programme [78], focusing on coping mechanisms for disease-related stress and uncertainty. This could be integrated into regular team training sessions [79]. Peer support networks could be established, leveraging the team dynamics to create a culture of openness about mental health. To address potential stigma associated with mpox, educational programmes should be implemented, emphasizing factual information and dispelling myths. While advanced technologies such as AI-driven chatbots for mental health support may not be immediately implementable in many contexts, especially in developing countries, it is crucial to consider more accessible and cost-effective alternatives [80]. For instance, establishing a network of trained peer support volunteers within sports organizations could provide initial mental health triage and guidance to appropriate resources. Collaboration with local mental health professionals, including sports psychologists and psychiatrists familiar with infectious disease-related trauma, could help develop tailored interventions for athletes dealing with pandemic-related anxiety or depression [81]. These strategies, while ambitious, are designed with long-term preparedness in mind, recognizing that implementation may vary significantly based on resources and infrastructure available in different regions [82, 83].

### Vaccination strategies

Vaccination strategies for mpox in football face significant challenges, largely stemming from the contentious experiences with COVID-19 vaccines [84]. The football community’s hesitancy is deeply rooted in the aftermath of the previous pandemic, where widespread concerns about vaccine side effects and long-term impacts emerged [81]. During the COVID-19 vaccination campaigns, reports of adverse events among athletes garnered significant attention. Cases of myocarditis, particularly in young male athletes, sparked intense debate and concern [85]. While rare, these incidents were highly publicized, leading to amplified fears within the sports community [86]. Some players reported experiencing fatigue, reduced performance, and prolonged post-vaccination recovery times, further fuelling scepticism [87].

The rapid development and emergency authorization of COVID-19 vaccines led to concerns about insufficient long-term safety data [88]. This worry is particularly acute among athletes, whose careers depend on peak physical condition and longevity [88]. The notion of introducing a novel medical intervention into finely tuned athletic bodies has met resistance from players and medical staff alike [89]. Conspiracy theories and misinformation, which spread rapidly through social media and even among some high-profile athletes, have left a lasting impact. Claims about vaccines affecting fertility, altering DNA, or being part of larger control agendas, though debunked, continue to resonate with a segment of the population, including some in the football world [90].

The perceived pressure to vaccinate during the COVID-19 pandemic, sometimes linked to ability to play or travel, led to feelings of coercion among some athletes [91, 92]. This has created a backdrop of mistrust towards health authorities and team management when it comes to vaccination policies [93]. Reports of breakthrough infections among vaccinated individuals during the COVID-19 pandemic have led some to question the efficacy of vaccines altogether. This scepticism may carry over to mpox vaccines, particularly if early data show anything less than perfect protection [94]. Moreover, the evolving nature of COVID-19 vaccines, with multiple boosters becoming necessary, has led to fatigue and scepticism about the long-term commitment required for vaccination programmes [94, 95].

The global disparity in vaccine access during the COVID-19 pandemic also created tensions, particularly in international football, where players from different countries had vastly different experiences with vaccine availability and mandates [96]. These compounding factors from the COVID-19 experience present a formidable challenge for potential mpox vaccination strategies in football. The memory of adverse events, the spread of misinformation, and a general wariness towards rapidly developed vaccines create a landscape where achieving high vaccination rates may be significantly more difficult than anticipated [97]. This hesitancy could potentially undermine efforts to establish effective herd immunity within the football community, leaving the sport vulnerable to mpox outbreaks [97]. To address these challenges, a multi-faceted approach is necessary, as outlined in Table 3.

**TABLE 3:** Vaccination strategies for a potential mpox pandemic in sports.

Strategy	Implementation	Expected outcome
Tailored communication	Athlete-specific messaging Use player ambassadors Share personal stories	Increased vaccine acceptance through peer influence
Address specific concerns	Studies on performance effects Clear safety information Transparent reporting	Alleviation of fears about performance and health impacts
Cultural sensitivity	Work with cultural leaders Culturally appropriate info Personal choice framework	Improved acceptance among diverse player groups
Alternative protocols	Robust testing for unvaccinated Incentives for vaccinated	Maintained team safety while encouraging vaccination
Long-term monitoring	Ongoing health monitoring Transparent data sharing	Address concerns about future effects, demonstrate player welfare commitment

It is important to note that while we propose various advanced technological solutions, including AI-driven mental health support, many of these recommendations may not be feasible in the immediate future, especially in resource-limited settings [98]. This framework is designed not only for potential mpox outbreaks but also as a long-term preparedness strategy for future pandemics that may emerge in the coming decades [99]. The implementation of these measures should always consider the diverse economic realities across different countries and aim for equitable, adaptable solutions [99].

## POTENTIAL IMPACT OF MPOX ON SPORTS ACTIVITIES

### Physical health considerations

Mpox infection bring about particular concerns to athletes’ physical health, distinct from those observed with COVID-19. The primary physical health considerations include the impact of skin lesions on performance, systemic effects on endurance, and potential long-term consequences [100]. Skin lesions, a hallmark of mpox, can significantly impair athletic performance, especially in sports requiring extensive skin contact or equipment use. These lesions may affect grip strength, dexterity, and mobility, factors crucial for many sports. Moreover, the risk of secondary bacterial infections in lesions may increase due to sweating and physical contact inherent in sports activities [17].

The systemic effects of mpox, including fever, fatigue, and muscle aches, can severely influence an athlete’s endurance and overall physical capacity [17]. Lymphadenopathy, typical of mpox, may affect mobility and comfort, particularly in sports requiring a full range of motion. While less prominent than in COVID-19, respiratory symptoms can occur, potentially affecting cardiovascular endurance and performance in aerobic sports [3, 17].

Dehydration and thermoregulation present additional concerns. The fever associated with mpox, combined with physical exertion, may increase the risk of heat-related illnesses. Athletes may need to pay extra attention to hydration and cooling strategies during recovery. Nutritional challenges may arise due to potential appetite loss during infection, leading to unintended weight loss and muscle wasting [101]. This necessitates careful nutritional management during and after recovery to maintain optimal body composition and performance. Indeed, long-term physical effects of mpox in athletes remain largely unknown [17]. Potential concerns include scarring from lesions, which could affect sports with aesthetic components. Possible lingering fatigue or weakness may require extended rehabilitation periods. The stress on the immune system may also leave athletes more susceptible to other infections or injuries in the short term after recovery [102].

While less common than in COVID-19, cardiovascular complications should be monitored, especially given the high cardiovascular demands of many sports. Rare ophthalmological involvement could have significant implications for sports requiring precise vision and eye-hand coordination [103].

The physical health considerations of mpox in athletes accentuate the need for sport-specific medical protocols and individualized assessment. Gradual return-to-play strategies, taking into account the unique demands of each sport and the specific manifestations of the disease in each athlete, will be crucial.

### Mental health impact

The mental health consequences of mpox for athletes result in particular difficulties, unlike other infectious diseases, particularly due to the visible nature of its symptoms and the potential stigma associated with the infection [104]. These factors could significantly affect an athlete’s psychological well-being, self-image, and overall mental state.

The presence of visible skin lesions due to mpox can be particularly distressing for athletes, who often have a heightened awareness of their physical appearance. This visible manifestation of the disease may lead to increased anxiety, depression, and self-consciousness [3]. Athletes in sports with aesthetic components or those who rely heavily on sponsorships and public image may experience amplified psychological distress. Stigma and misconceptions surrounding mpox can exacerbate mental health challenges [105]. The initial association of the 2022 outbreak with specific communities may lead to unwarranted discrimination or prejudice. Athletes may fear social isolation or judgment from teammates, competitors, or the public, potentially affecting their social support systems and overall well-being [106].

The uncertainty surrounding the long-term effects of mpox on athletic performance can be a significant source of stress and anxiety. Athletes may worry about their ability to return to pre-infection performance levels, potentially leading to feelings of frustration, helplessness, or loss of identity tied to their athletic capabilities [107]. Isolation periods required for infection control can contribute to feelings of loneliness and a sense of disconnect from the team. Being away from regular training and competition environments may exacerbate existing mental health symptoms/issues or introduce new ones, particularly for athletes who rely heavily on a structured schedule and routines for their psychological well-being [33, 106].

The potential impact on career trajectories, especially for athletes in time-sensitive competitive phases or nearing the end of their careers, can lead to heightened stress and anxiety. Concerns about missed opportunities, contract negotiations, or selection for major competitions may weigh heavily on infected athletes [108].

Coping with the physical symptoms of mpox, including fever, fatigue, and pain from skin lesions, can also take a psychological toll. The discomfort and potential pain associated with the infection may lead to mood disturbances, irritability, and decreased motivation. A well-prepared medical staff should support the athletes in managing these issues.

### Performance implications and training adaptations during potential infection, quarantine or lockdown

The impact of mpox on athletic performance and the necessary training adaptations present unique challenges that differ significantly from those encountered during the COVID-19 pandemic [17]. Mpox infection creates particular challenges to athletic performance, necessitating careful consideration of both immediate and long-term impacts on training and competition readiness. In the immediate aftermath of infection, athletes may experience significant deconditioning due to the systemic effects of mpox, including fever, fatigue, and muscle pain [109]. This deconditioning can lead to reductions in aerobic capacity, strength, and sport-specific skills. However, the presence of skin lesions introduces an additional layer of complexity to performance management and training adaptations compared to COVID-19 for instance [110].

Indeed, skin lesions can substantially impair an athlete’s ability to perform, especially in sports requiring extensive skin contact or fine motor control [111]. These lesions may cause discomfort or pain during movement, potentially altering biomechanics (technique) and increasing the risk of secondary injuries. Moreover, the psychological impact of visible lesions may affect an athlete’s confidence and mental readiness to return to training and competition [112].

Training prescriptions for mpox-infected athletes must be carefully tailored to account for these skin-related challenges. Low-impact cardiovascular exercises that minimize friction on affected skin areas would become crucial. For instance, stationary cycling or rowing may be preferable to running or contact sports during the recovery phase [17]. Strength training protocols need modification to avoid putting pressure on lesions, potentially emphasizing isometric exercises or exercises that target unaffected body parts [17].

Flexibility and mobility work take on heightened importance in the context of mpox recovery. Skin lesions may lead to stiffness or a restricted range of motion in affected areas [17]. Gentle stretching and mobility exercises, carefully executed to avoid irritating healing lesions, could help maintain joint flexibility and prevent the development of compensatory movement patterns that might lead to future injuries [109].

The application of high-intensity interval training (HIIT) in mpox recovery requires careful consideration [113]. While HIIT proved valuable during COVID-19 lockdowns for maintaining cardiovascular fitness, its intense nature may exacerbate skin lesions or cause excessive discomfort in mpox patients [113]. A modified approach to interval training, featuring lower intensities and shorter durations, may be more appropriate. As recovery progresses, the intensity and complexity of these sessions can be gradually increased, always with close monitoring of skin condition and overall health status.

The reintroduction of sport-specific training must be approached with caution, particularly in sports involving skin-to-skin contact or shared equipment. Modified drills that limit skin contact should be prioritized in the early stages of return to play [114]. For example, in combat sports, shadow boxing or solo technique work might precede partner drills. In team sports, individual skill work might be emphasized before full team practices resume. Continuous monitoring of skin healing and overall health status is crucial throughout the recovery and return-to-play process. This monitoring should inform a gradual progression in training intensity and complexity. The use of protective gear or specialized clothing may be necessary to shield healing skin during training and early return to competition [115].

The unique challenges presented by a potential mpox outbreak highlight the need for a flexible, individualized approach to athlete management [17]. Sports organizations must develop comprehensive protocols that address not only the physical aspects of recovery, but also the psychological impact of the disease [33]. These protocols should be adaptable, recognizing that the manifestation and recovery from mpox can vary significantly among individuals [17]. As the sports medicine community continues to learn about the long-term effects of mpox on athletic performance, ongoing research and data collection will be crucial [17, 116]. Professional sports science and sports medicine societies such as the European College of Sport Science and the American College of Sports Medicine should feature symposia on mpox at their annual meetings. This knowledge will inform the development of more refined strategies for maintaining athlete health and performance in the face of this emerging infectious disease threat [17].

**FIG. 2:**
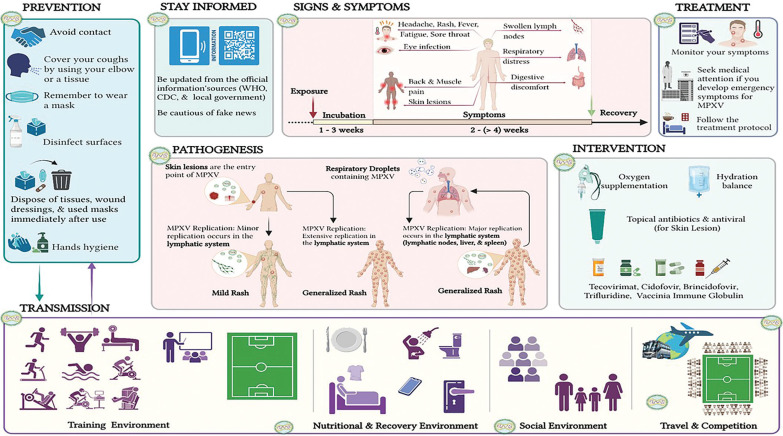
Comprehensive overview of mpox virus (MPXV) transmission, symptoms, pathogenesis, prevention, and management in football players’ environments (created with Biorender.com) [117-122]. CDC: Centers for Disease Control and Prevention. WHO: World Health Organization. The figure provides a comprehensive overview of mpox transmission, clinical symptoms, pathogenesis, and management, with a specific focus on football players and sports environments. Transmission pathways include horizontal transmission through direct human-to-human contact and indirect transmission from contaminated environments and surfaces. Football players face specific risks due to skin-to-skin contact during match play or training, respiratory droplet transmission in close quarters (e.g. locker rooms, restaurants), and indirect/fomite transmission via shared sports equipment, clothing, and surfaces. Prevention and safety measures are crucial in mitigating the spread of mpox in sports settings. These include isolating infected individuals, using PPE, implementing proper hygiene practices, disinfecting shared equipment, and regularly sanitizing sports facilities. Reliable information on mpox is crucial. Local government agencies, the CDC and the WHO provide up-to-date, evidence-based guidance on transmission, prevention, and management of this disease. Athletes, coaches, and sports organizations should regularly consult these official sources for the latest developments and best practices. Avoiding misinformation is essential for appropriate response and prevention strategies. The clinical symptoms of mpox progress from initial symptoms such as fever, fatigue, and lymphadenopathy to the development of a skin rash that spreads across the body. These symptoms can significantly influence athletic performance and participation. The pathogenesis of mpox in athletes involves viral invasion and replication in the body, leading to systemic and localized symptoms. Skin lesions and fatigue can particularly affect physical endurance and overall athletic performance. Emergency clinical support and treatment. Athletes developing symptoms of mpox should seek immediate medical attention. Emergency clinical support may include isolation to prevent further transmission, symptomatic treatment, and oxygen supplementation if respiratory symptoms are severe, addressing dehydration through fluid replacement, administration of antivirals in severe cases, and use of vaccinia immunoglobulin for complications or severe infections. For skin lesions, topical antibiotics may be recommended to prevent secondary bacterial infections. Antiviral medications may also be applied topically in some cases. Healthcare professionals based on the individual’s symptoms, medical history, and disease severity should determine the specific treatment plan. Sports medicine physicians should work closely with infectious disease specialists to provide appropriate care for athletes with mpox while considering the unique needs of sports participation. Understanding the transmission, prevention, and clinical aspects of mpox is essential for maintaining the health and safety of football players, other athletes and the support staff surrounding them. This comprehensive guide is based on research by some authors and organizations. It provides valuable information for understanding mpox’s impact on football players and athletes, as well as strategies for prevention and management in sports environments.

Figure 2 presents a comprehensive overview of mpox transmission, symptoms, pathogenesis, prevention, and management in football players’ environments [117–122].

## MANAGEMENT STRATEGIES FOR SPORTS EVENTS

### Risk assessment for event continuity

The process of risk assessment for sports events in the context of mpox necessitates a comprehensive and nuanced approach, building upon the valuable global experiences gained during the COVID-19 pandemic [25, 123, 124]. This assessment requires the collaboration of a multidisciplinary team comprising health experts, event organizers, and sports’ governing bodies to thoroughly evaluate the potential risks and benefits associated with holding an event [125, 126].

The risk assessment process must consider several key factors, including the current epidemiological situation in both the host location and participating countries, the nature of the sport and the level of physical contact involved, the duration and scale of the event, the availability and effectiveness of preventive measures, and the capacity of local healthcare systems to manage potential outbreaks [127]. These considerations should be weighed carefully against the potential benefits of hosting the event, such as economic impact, social cohesion, and the continuation of sporting traditions.

The Amir Cup 2020 in Qatar serves as a pioneering example of event management during a pandemic [124]. Held at a time when there was no availability of vaccines and with the presence of 20 thousand spectators, it demonstrated the feasibility of safe event execution through the implementation of rigorous testing protocols, strict hygiene measures, and carefully controlled spectator numbers (half of the stadium capacity) [128, 129]. This approach provides a valuable template that can be adapted to address the unique challenges presented by mpox, with additional considerations given to the risks associated with skin-to-skin contact and shared equipment use.

### Adaptation vs. cancellation protocols

The decision-making process regarding whether to adapt or cancel a sports event due to mpox should be rooted in a thorough risk-benefit analysis. This analysis must be informed by the latest scientific understanding of the disease, its transmission dynamics, and the effectiveness of available preventive measures [130]. The experiences of major sporting events during the COVID-19 pandemic offer invaluable insights into this complex decision-making process [130].

Adaptation strategies may encompass a wide range of modifications to traditional event formats and procedures. These could include altering competition structures to minimize close contact between athletes, implementing enhanced hygiene protocols and regular health screenings, utilizing controlled “bubble” environments as successfully demonstrated by the National Basketball Association (NBA) and National Hockey League (NHL) during the height of the COVID-19 pandemic, and adjusting event schedules to accommodate potential isolation periods if required [131].

The Union of European Football Associations (UEFA) Europa League’s completion in a reduced format in 2020 stands as a testament to the feasibility of adapting major tournaments in the face of infectious disease threats [132]. Similarly, the Tokyo 2020 Olympics, postponed to 2021, exemplified how large-scale, multi-sport events could be conducted safely with the introduction of vaccines, albeit without spectators. These examples provide valuable blueprints for potential adaptations in the context of mpox [133].

Cancellation of an event should be considered as a last resort, to be implemented only when the associated risks demonstrably outweigh the benefits and cannot be adequately mitigated through adaptations. It is crucial to establish clear, predetermined thresholds for cancellation based on objective criteria such as infection rates, the ability to maintain a safe environment for all participants, and the potential for the event to become a vector for disease spread.

### Spectator management

The management of spectators in the context of mpox requires a carefully calibrated approach that balances public health imperatives with the desire for fan engagement and the economic considerations of event organizers [134]. The gradual reintroduction of spectators during the later stages of the COVID-19 pandemic provides a valuable template for managing this critical aspect of sports events [97, 135].

Effective spectator management strategies may include a phased reintroduction of fans, beginning with reduced capacities and gradually increasing as the epidemiological situation allows. This approach should be coupled with robust health screening measures at entry points, enhanced cleaning and disinfection protocols, particularly for high-touch surfaces, and potential modifications to seating arrangements to maintain appropriate physical distancing [128]. The implementation of PPE for staff and recommendations for spectator use may also be necessary, depending on the current understanding of mpox transmission dynamics [136].

The progression from the Amir Cup 2020, held with limited spectators and stringent protocols, to the FIFA World Cup 2022 in Qatar, which successfully hosted full stadiums, illustrates the potential for a gradual return to normalcy. The World Cup demonstrated how large-scale events could be held safely, relatively shortly after the global COVID-19 pandemic [137]. This was possible by leveraging high vaccination rates and the development of herd immunity. However, it is important to note that the approach to mpox may initially need to be more conservative, given the different transmission characteristics of the disease and the current lack of a widely available specific vaccine for the general population [129, 137].

The successful management of sports events in the face of the mpox threat will require a high degree of flexibility, innovation, and an unwavering commitment to prioritizing the health and safety of all participants and spectators. By carefully adapting the lessons learned from the COVID-19 pandemic to the specific challenges caused by mpox, sports organizations can work towards maintaining the continuity of events while effectively minimizing health risks [32].

### FIFA World Cup 2026: epidemiological preparedness in the context of emerging mpox threats

The forthcoming FIFA World Cup 2026, to be hosted across Canada, Mexico, and the United States, presents a complex epidemiological challenge, particularly in light of recent mpox developments and insights gleaned from the Paris 2024 Olympics. As of this writing, just a few weeks after the conclusion of the Paris Games, the global health community finds itself in a state of heightened vigilance regarding the mpox situation.

It is crucial to note that while the situation in Paris did not escalate to pandemic proportions during the Olympics, the potential for future escalation remains a significant concern. The identification of a more virulent strain, Clade 1b, in the immediate post-Olympic period has raised alarms about the possibility of a more widespread outbreak in the coming months or years [138]. This temporal proximity to the Paris Games underlines the dynamic nature of infectious disease threats and the need for continual reassessment of epidemiological strategies.

The tri-national nature of the 2026 World Cup in North America introduces unique challenges in epidemiological harmonization. The event’s vast geographical scope, spanning diverse ecological and climatic zones from Mexico to Canada, necessitates a comprehensive approach to disease surveillance and control. This approach must account for the potential variability in mpox transmission dynamics across different environments and population densities.

Lessons from Paris 2024, while not directly involving a full-scale pandemic response, provide valuable insights into managing large-scale international events under the shadow of emerging infectious threats. The Paris experience demonstrated the importance of robust syndromic surveillance systems, rapid molecular diagnostics, and flexible public health measures [139]. However, the post-Olympic emergence of Clade 1b highlights the need for even more advanced genomic surveillance and real-time phylodynamic analysis capabilities for future events [140].

The North American World Cup organizers should prepare for a scenario in which the current mpox situation could potentially evolve into a more serious global health threat by 2026. This necessitates the development of scalable response plans that can adapt to various levels of disease prevalence, from isolated clusters to widespread community transmission. The implementation of a continental-scale genomic surveillance network, capable of rapidly identifying and characterizing new viral variants, will be crucial [141].

Risk assessment and communication strategies for the 2026 World Cup must be designed with the understanding that the global mpox landscape may be significantly different from the present. This includes preparing for the possibility of a pandemic-level event, even if such a scenario has not yet materialized. The development of adaptive threshold criteria for implementing various levels of public health interventions, based on real-time epidemiological data, will be essential [142].

The potential for mpox to affect a broader demographic by 2026, beyond the currently observed high-risk groups, must be considered in all preparedness plans. This shift in epidemiology, if it occurs, would necessitate a re-evaluation of targeted screening and prevention strategies [143].

## ECONOMIC IMPACT AND MITIGATION STRATEGIES

The potential resurgence of mpox, particularly in the context of the global sports calendar, presents a multifaceted economic challenge that extends far beyond individual events. The interconnected nature of the international sports ecosystem, encompassing events such as the FIFA World Cup 2026, various continental and domestic football leagues, and numerous other sports competitions worldwide, necessitates a comprehensive analysis of the potential economic impact and the development of robust mitigation strategies [25].

The financial implications for the global sports industry are profound and far-reaching. The emergence of more virulent mpox strains, such as Clade 1b, introduces a level of uncertainty that permeates all aspects of sports economics. Revenue streams across multiple events and leagues could face potential disruption, with the possibility of crowd restrictions, schedule alterations, or, in extreme cases, event cancellations [17]. This uncertainty extends to broadcasting rights, sponsorship deals, and merchandise sales, potentially leading to a sector-wide re-evaluation of the financial models underpinning professional sports.

Major football tournaments (e.g. the 2025–26 UEFA Champions League) may face significant revenue challenges if mpox outbreaks necessitate operational changes. The impact could cascade through domestic leagues worldwide, from Europe’s top-tier competitions to emerging markets in Asia and Africa, potentially destabilizing club finances, player transfer markets, and wage structures [144]. The 2026 Winter Olympics in Milan-Cortina presents its own set of challenges, given the close-contact nature of many winter sports and the indoor environments of several venues. Any restrictions or outbreaks could severely affect not only the event’s direct revenues but also the long-term economic benefits to the host regions. It is important to note that while mpox has historically been endemic to certain regions of Africa, recent outbreaks have shown potential for global spread through international travel and contact [145].

The potential impact on broadcasting rights and media contracts, which are primary revenue sources for many sports, could be substantial. This could lead to complex liability issues and financial challenges across the sports industry [146]. Insurance and liability considerations in this multi-sport context become increasingly complex. The insurance landscape for sports events will likely undergo significant changes to account for the broadened risk profile. Event organizers across different sports may find it increasingly challenging to secure comprehensive coverage, particularly for communicable disease risks. Multi-sport organizations such as the International Olympic Committee and global governing bodies such as FIFA and UEFA may need to consider creating their own insurance pools or self-insurance mechanisms to manage the increased risk across their portfolio of events [147].

Liability issues become more nuanced when considering the variety of sports and their differing risk profiles. Contact sports may face higher liability risks compared to non-contact sports, potentially leading to a tiered approach in legal and insurance considerations. The global nature of sports tourism adds another layer of complexity, with organizers potentially needing to consider liability not just for the events themselves, but also for associated travel and accommodations [148].

Developing economic resilience in this complex, multi-sport environment requires a coordinated, adaptable approach. Cross-sport collaboration becomes crucial, with the potential establishment of a global sports resilience forum where different sports governing bodies can share best practices, coordinate responses, and potentially pool resources for research and development of safety measures [149]. This collaborative approach could lead to the development of standardized protocols that can be adapted to the specific needs of different sports and events.

Flexible scheduling and format innovations will be essential in navigating the uncertain landscape. Sports organizations must develop adaptive competition formats that can be quickly implemented across various sports [150]. This could include provisions for regional bubbles, extended but less intensive schedules, or hybrid in-person/virtual events. Such flexibility will be crucial in maintaining competitive integrity and financial viability in the face of potential disruptions [151].

Diversification of revenue models emerges as a key strategy for economic resilience. This includes accelerating the development of virtual and augmented reality experiences across all sports, creating new digital assets, and exploring novel competition formats that are less reliant on physical attendance [152]. The integration of technology not only serves as a risk mitigation strategy but also opens up new avenues for fan engagement and monetization [153].

Investment in biosecurity infrastructure becomes a priority, with the development of multi-use venues featuring advanced health screening capabilities, adaptable space configurations, and state-of-the-art ventilation systems.

## CONCLUSIONS

This comprehensive analysis of the potential impact of an mpox outbreak on the sports world, with a particular focus on football, offers a proactive framework for addressing emerging infectious disease threats in athletic settings. Mpox may not be a pandemic today (as of late September 2024), but neither was COVID-19 when it first appeared. The real question is not “Why study this now?” but rather, “What happens if we do not?” The COVID-19 research was not just about understanding the virus, but also about preparedness. It helped us manage the crisis, but, more importantly, it taught us that early action could prevent worse scenarios. The time to act is before a crisis, not when it is too late. This research represents a forward-thinking effort to anticipate and prepare for potential challenges, using the valuable lessons learned from the COVID-19 pandemic.

The study’s approach aligns with the call for action in sports epidemiology, encouraging the scientific community to gather and share data on post-event infection rates in large sporting events. We highlight the distinct characteristics of mpox compared to COVID-19, underlining the need for tailored strategies in sports settings. By examining the unique risks caused by close-contact sports such as football, this research proposes innovative approaches to risk assessment, including the potential use of COVID-19 specific applications for health tracking, and suggests tailored testing protocols and hygiene measures that could be implemented proactively.

A key strength of this framework is its foundation in the experiences gained from managing COVID-19 in sports. Unlike the situation faced at the onset of the COVID-19 pandemic, the sports world is now better equipped with knowledge, infrastructure, and adaptive strategies to handle potential global health threats. This preparedness allows for more nuanced and effective approaches to issues such as spectator management, international coordination, and mental health support. Further, the limitations observed in previous research on mpox transmission, such as the lack of information on the type of person-to-person contact and the potential bias introduced by post-exposure prophylaxis and vaccination, have been avoided. We have also addressed the critical aspect of vaccination strategies, acknowledging the challenges of vaccine hesitancy stemming from the COVID-19 experience. By recognizing these challenges and constraints’’, sports early, sports organizations can develop (i) communication that is more effective and (ii) prevention implementation plans.

## Declaration

The authors wish to disclose that an artificial intelligence tool (ChatGPT 3.5) was utilized to enhance the clarity and coherence of the manuscript writing. The tool was used for language refinement purposes only, ensuring that the text was clear and coherent without altering the scientific content or generating any new text [154, 155].
